# Immune Activation Reduces Sperm Quality in the Great Tit

**DOI:** 10.1371/journal.pone.0022221

**Published:** 2011-07-12

**Authors:** Sylvain Losdat, Heinz Richner, Jonathan D. Blount, Fabrice Helfenstein

**Affiliations:** 1 Evolutionary Ecology Lab, Institute of Ecology and Evolution, University of Bern, Bern, Switzerland; 2 Centre for Ecology and Conservation, College of Life and Environmental Sciences, University of Exeter, Penryn, United Kingdom; Arizona State University, United States of America

## Abstract

Mounting an immune response against pathogens incurs costs to organisms by its effects on important life-history traits, such as reproductive investment and survival. As shown recently, immune activation produces large amounts of reactive species and is suggested to induce oxidative stress. Sperm are highly susceptible to oxidative stress, which can negatively impact sperm function and ultimately male fertilizing efficiency. Here we address the question as to whether mounting an immune response affects sperm quality through the damaging effects of oxidative stress. It has been demonstrated recently in birds that carotenoid-based ornaments can be reliable signals of a male's ability to protect sperm from oxidative damage. In a full-factorial design, we immune-challenged great tit males while simultaneously increasing their vitamin E availability, and assessed the effect on sperm quality and oxidative damage. We conducted this experiment in a natural population and tested the males' response to the experimental treatment in relation to their carotenoid-based breast coloration, a condition-dependent trait. Immune activation induced a steeper decline in sperm swimming velocity, thus highlighting the potential costs of an induced immune response on sperm competitive ability and fertilizing efficiency. We found sperm oxidative damage to be negatively correlated with sperm swimming velocity. However, blood resistance to a free-radical attack (a measure of somatic antioxidant capacity) as well as plasma and sperm levels of oxidative damage (lipid peroxidation) remained unaffected, thus suggesting that the observed effect did not arise through oxidative stress. Towards the end of their breeding cycle, swimming velocity of sperm of more intensely colored males was higher, which has important implications for the evolution of mate choice and multiple mating in females because females may accrue both direct and indirect benefits by mating with males having better quality sperm.

## Introduction

The immune system plays a pivotal role in defending an animal against attacks by parasites and pathogens but it also has fitness-related costs. Costs have been reported for survival [1], [2], reproductive output [3], [4], [5], and growth [6], [7]. Mounting an immune response is energetically demanding [8] and it is generally believed that the cost of immunity results from energy being diverted away from reproductive functions and/or somatic maintenance thus reducing reproductive success and/or survival [2]. However, there is also evidence that antioxidants are an important resource, which can be limiting for immune function [9]. Indeed, oxidative stress arising from energy turnover could be a key mechanism to explain the costs of immunity [10], [11].

Oxidative stress is the imbalance between pro- and antioxidants in favor of the former [12]. Organisms experience such stress when a sudden increase in pro-oxidants overwhelms their antioxidant defenses, which can be the case when the immune system is activated. Within a few hours following an infection, vertebrate organisms mount an innate, non-specific inflammatory response during which immune cells such as phagocytes (e.g. granulocytes, macrophages) are recruited to the focal site. Phagocytes use highly reactive oxygen and nitrogen species (ROS and RNS) to kill engulfed pathogens and to regulate inflammation [13]. Although ROS and RNS are effective antimicrobial agents, they may generate local and systemic oxidative stress and cause oxidative damage to the host organism [14].

Vertebrate spermatozoa are highly susceptible to oxidative stress due to the high proportion of polyunsaturated fatty acids in their membrane and to their highly condensed DNA and reduced transcription machinery that limits DNA repair [15]. Oxidative damage to spermatozoa can reduce fertility in domestic animals and humans [15], [16], and has been demonstrated recently in a wild bird species to affect sperm quality by a reduction of sperm motility and swimming velocity [17]. Remarkably, immune-induced systemic oxidative stress has been found also to reduce male fertility in humans [16], but whether immune-induced oxidative stress can result in oxidative damage to sperm and reduced sperm quality in non-human species still remains unexplored.

The negative impact of oxidative damage to sperm can have dramatic consequences for male fitness because an important part of a male's reproductive success depends on his fertilization potential and likelihood of paternity [18]. In species where males face sperm competition, the circumstance when ejaculates of different males compete to fertilize a common set of ova, it is now well established that male fertilization potential and paternity depend both on sperm numbers (i.e. ejaculate size and percentage of motile sperm) and sperm quality (i.e. sperm swimming velocity and longevity) [19], [20], [21], [22], [23].

Recently, Chargé et al. [24] demonstrated in a captive bird species, the houbara bustard *Chlamydotis undulata undulata*, that mounting an immune response entails a cost in terms of reduced sperm quality and reproductive success. In the present study, we aimed at testing the hypothesis that activating the immune system may lead to a reduction in sperm quality through systemic oxidative stress and oxidative damage to sperm. We thus immune challenged a group of males with lipopolysaccharide (LPS), an epitope of gram-negative bacteria, while a second group was injected with phosphate buffer saline (PBS) to serve as a control. LPS induces an inflammatory response and mimics a bacterial infection without producing the cost of the pathogen itself, thus enabling to address the cost of immunity alone [4]. Antioxidant compounds such as superoxide dismutase (SOD), glutathione (GSH), vitamin C and vitamin E have been found in avian semen [25], [26]. Vitamin E is a non-pigmentary lipophilic antioxidant that animals must obtain from their diet, which was shown to have a major role in protecting spermatozoa from oxidative damage [26]. Therefore, we crossed our immune-challenge with a vitamin E supplementation to test whether vitamin E protects sperm against immune-induced oxidative stress. Finally, carotenoid-based traits have been proposed to signal male antioxidant capacity [27] and particularly the ability to find, absorb and use non-pigmentary antioxidants [28], [29]. Moreover, carotenoid-based plumage traits have recently been found to signal male ability to protect sperm against oxidative stress [17]. Thus, we also measured male carotenoid-based coloration. We used the great tit *Parus major*, a sexually dimorphic species [30] in which males exhibit yellow, carotenoid-based breast plumage. Additionally, sperm competition is significant in this species with up to 50% of the males losing some paternity [31]. Thus, sperm quality and male ability to protect sperm from oxidative stress are likely major determinants of male reproductive success. Our fully crossed 2×2 design aimed at testing whether mounting an immune response 1) reduces sperm quality and 2) reduces somatic antioxidant capacity and increases levels of oxidative damage to sperm and soma, two indices that are expected to predict oxidative stress. Additionally, we tested 3) whether vitamin E participates in the antioxidant protection of sperm and thus alleviates the cost of mounting an immune response, and 4) whether carotenoid-based coloration reflects a male's sperm quality.

## Materials and Methods

### General procedure

This experiment was carried out during spring 2009 in a natural population of great tits breeding in nest boxes in a forest near Bern, Switzerland (46°7′N, 7°8′E). Nest boxes were regularly visited from the beginning of the breeding season to determine in 67 nests the start of egg laying and hatching dates. Seven days post-hatch, we captured all 67 males at the nest using clap nets triggered from a distance using a nylon string. We measured their body mass (±0.1 g), tarsus length (±0.05 mm), took a blood sample from the brachial vein, took two sperm samples, performed both immune challenge and vitamin E treatments and recorded plumage reflectance (see below). On day 13 post-hatch all males were recaptured and blood-sampled and sperm sampled again. In both catching occasions, we used 7 µl of whole blood to analyze red blood cell resistance to oxidative stress (KRL test) and 5 µl of plasma (after centrifugation) to measure lipid peroxidation (malondialdehyde, MDA levels).

This study was approved by the Ethical Committee of the Agricultural Office of the Canton Bern, Switzerland (experimentation permit 118/07) and the Federal Agency for Environment of the Canton Bern, Switzerland (ringing permit 2739).

### Immune challenge

By tossing a coin males were randomly assigned to be injected in the wing web with either 0.01 mg of Lipopolysaccharide (LPS, *E. coli*, serotype: 055:B5, Sigma-Aldrich, Basel, Switzerland) diluted in 0.02 ml of PBS (Phosphate Buffer Saline: 0.5 mg/ml; ca. 0.5 mg of LPS per kg of body mass) or with the same volume of PBS (0.02 ml) only.

### Vitamin E supplementation

In addition to the immune challenge and according to a fully randomized, full-factorial design, males were force-fed with either one fresh, living *Calliphora spp.* larva coated with 1.84 mg of α-tocopherol acetate (Sigma-Aldrich, Basel, Switzerland) using corn oil as an excipient or with a larva coated with corn oil only. Vitamin E was provided once (on Day 7 post-hatch) in quantities aiming at doubling the daily amount great tits naturally obtain from their diet over the entire 6-day experimental period. These quantities were calculated according to the estimated daily food intake of great tits (DFI: 22.15 g of lepidopterian larvae per day [32] and the estimated concentration of vitamin E in their food (0.04±0.03 µmol of vitamin E per g of lepidopterian [33]). The relatively small amount of vitamin E supplemented was chosen because providing more than one standard deviation of the daily natural intake of Vitamin E may lead to saturation or to an absence of a positive effect [34].

All males included in the analyses did not differ significantly in their tarsus length, body mass and mate's laying date with regard to the four experimental groups (all F_1, 63_<1.54, p>0.22; Immune challenged/Vitamin E-supplemented: n = 19; Control/Vitamin E-supplemented: n = 15; Control/Placebo: n = 15; Immune challenged/Placebo: n = 18). LPS-injected males had a smaller brood size at hatching (mean ± sd; 8.5±1.6) than control (9.3±1.4) (F_1, 63_<5.31, p = 0.025).

### Breast color

On day 7 post-hatch, we recorded reflectance spectra of the yellow breast plumage on four different patches, i.e. on both sides of the keel, on the furcula and on the belly. We took two reflectance readings per patch to assess repeatability, removing the probe from the plumage between each measure. Spectral measures were made using a USB4000 spectrophotometer, an FCR-7UV200-2-ME bifurcated reflectance probe with a 200 µm fiber core diameter, and a deuterium-halogen/tungsten light source (DH-2000-BAL, UV-VIS-NIR; Ocean Optics Inc., Netherlands). Measures were made following the recommendations by Andersson and Prager [35]. The tip of the probe was fitted with a black PVC cylinder to standardize measuring distance and exclude ambient light. The probe was held perpendicular to the plumage surface. Each measurement was the average of four scans with a 100 ms integration time, and was calculated relative to a diffuse reflectance standard (WS-1, Ocean Optics Inc., Netherlands). The spectrophotometer was calibrated before each individual was measured. SL took all measurements.

Color vision in birds depends on four types of single cones that are sensitive to very short (VS), short (S), medium (M), and long (L) wavelengths [36]. Recently, physiological models of color vision have been developed [37], [38], which allow to describe a colored trait in the eye of a conspecific taking into account the spectral sensitivity of the retinal cones, the transmittance properties of the ocular media and the ambient light irradiance spectrum [38]. Using the SPEC package (http://www.bio.ic.ac.uk/research/iowens/spec/welcome.htm; [39]), we computed four cone quantum catches that quantify the amount of light captured by each of the avian single cones [37]. We used data on cone spectral sensitivities and ocular media transmittance for the blue tit *Cyanistes caeruleus* [36]. Passerine species that are sensitive to ultraviolet wavelength show little variation in their spectral sensitivity, and using the average cone-capture function for 11 UV-type species provided by Endler et al. [38] did not qualitatively change the results. We used the forest shade irradiance spectrum [40] because our great tit population breeds in forest. The cone catches were standardized using the von Kries algorithm to account for color constancy [36]. Each cone quantum catch was divided by the sum of all four, and relative cone quantum catch were then transformed according to Kelber et al. [41]. This transformation projects the tetrahedral avian visual space into a three-dimensional space. Each color measure is now defined by a set of Euclidean x, y, z coordinates where higher values of x represent greater stimulation of the L-cones and lower stimulation of the M-cones, higher y values represent greater stimulation of the S-cones, and higher values of z represent greater stimulation of the VS-cones. The x, y and z coordinates were repeatable over the two repetitions per patch (intra-class correlation coefficients derived from random-effect models: x: r = 0.50, p<0.001; y: r = 0.49, p<0.001; z: r = 0.59, p<0.001). Measurements were thus averaged per repetition and further per patch to characterize each individual.

We then conducted a principle component analysis to characterize the variation in color in this Euclidian space [42]. The first principal component explained 90% of the variance and was positively correlated with x (r = 0.57) and negatively correlated with y (r = −0.57) and z (r = −0.59). Therefore, PCA1, hereafter referred to as “breast color”, reflects a variable ranking males from those with more yellow (positive scores) to less yellow plumage (more “green-blue”) (negative scores). This estimate of breast color is highly positively correlated with carotenoid chroma (r = 0.78, p<0.001, n = 67), a known measure of the amount of pigment deposited in the feathers [43], computed as (*R*_700_−*R*_450_)/*R*_average_.

### Sperm quality analyses

We performed the experiment at the nestling stage, a period when males can be easily captured at the nest when feeding their offspring. We believe that studying sperm quality at this stage is biologically relevant for two main reasons. First, although great tits are fairly synchronous, the breeding season stretches over several weeks so that some pairs may be at the laying stage while others are at the nestling stage. This may allow males to seek extra-pair copulations, a common phenomenon in this species [44] (e.g. 11.4% of extra-pair nestlings on average in our population). Sperm quality would then be crucial to accrue fitness benefits via extra-pair paternity throughout the whole breeding season. Second, great tits may produce second clutches [45] and sperm quality remains of importance to fertilize females and secure paternity in these broods. Hence, male great tits may be selected to maintain high sperm quality throughout the breeding season, which may explain why, despite the energetic cost they may incur [46], male great tits do no cease producing sperm during chick rearing.

Ejaculates (ca. 0.5 µl) were collected by gently massaging the male's cloaca [47] at the start of the experiment (7 days post-hatch) and six days later (13 days post-hatch) (n = 67). Sperm were immediately mixed with 50 µl of pre-warmed D-MEM (4500 mg glucose/l, 110 mg sodium pyruvate/l, 4 mM L-glutamine, Sigma Aldrich, Basel, Switzerland), and a 9 µl of sperm/buffer solution was immediately transferred under a microscope with dark-field condition and sperm motion video recorded for two minutes at 40°C. Sperm motion was analyzed after 0, 60, 120 seconds of recording using a CASA plug-in to Image J [48]. We recorded the percentage of motile sperm and mean values for VCL, VAP, VSL, linearity (VSL/VAP), wobble (VAP/VCL), BCF, progression (average distance from origin on the average path during all frames analyzed) and efficiency (progression/VAP). The number of sperm cells detected was recorded to account for sperm density in the sample. We assessed sperm quality as (1) the percentage of motile sperm and (2) Straight Line Velocity (VSL), hereafter referred as sperm swimming velocity. VSL was highly correlated with VCL and VAP (r = 0.92 and r = 0.98, p<0.0001, n = 64, respectively) and also with the PC1 scores from a principal component analysis of all variables measured excluding the percentage of motile sperm, as commonly used in some studies (r = 0.99, p<0.0001, n = 64). Because passerine spermatozoa are considered to travel in a straight line [49] and because VSL led to better fitted models while qualitatively giving the same results than VCL, VAP and PC1, we used it as a descriptor of sperm swimming velocity.

All measures were done blindly with respect to treatments by SL. Re-analysis of sperm motion videos immediately after ejaculate collection for all males sampled on day 7 yielded significant repeatability (intra-class correlation coefficients derived from random–effect models [50]) of our measures of sperm quality (percentage of motile sperm: r = 0.94, F = 212.9, p<0.0001, n = 66; sperm swimming velocity: r = 0.97, F = 305.7, p<0.0001, n = 66).

### Sperm lipid peroxidation

A second ejaculate (0.5–1.5 µl) was collected with a graduated 5-µl capillary tube, transferred into 10 µL PBS and frozen at −80°C until analyzed. Ejaculate concentrations of malondialdehyde (MDA), formed by the β-scission of peroxidized fatty acids, were assessed using HPLC with fluorescence detection, as described previously [51] with some modifications. All chemicals were HPLC grade, and chemical solutions were prepared using ultra pure water (Milli-Q Synthesis; Millipore, Watford, UK). Samples were first immersed in a water bath (ice cold) and sonicated for 10 minutes, then microtubes were homogenized for one minute using a motorized pestle before being centrifuged at 13,000 rpm and 4°C for 4 minutes. Sample derivitization was done in 2 ml capacity screw-top microcentrifuge tubes. To a 5 µl aliquot of sample or standard (1,1,3,3-tetraethoxypropane, TEP; see below) 5 µl butylated hydroxytoluene solution (0.05% w/v in 95% ethanol), 40 µl phosphoric acid solution (0.44 M), and 10 µl thiobarbituric acid (TBA) solution (42 mM) were added. Samples were capped, vortex mixed for 5 seconds, then heated at 100°C for exactly 1 hour in a dry bath incubator to allow formation of MDA-TBA adducts.

Samples were then cooled on ice for 5 minutes before 80 µl n-butanol was added and tubes were vortex mixed for 10 seconds. Tubes were then centrifuged at 13,000 rpm and 4°C for 4 minutes, before a 55 µl aliquot of the epiphase was collected and transferred to an HPLC vial for analysis. Samples (40 µl) were injected into a Dionex HPLC system (Dionex Corporation, California, USA) fitted with a 2 µm pre-column filter and a Hewlett-Packard Hypersil 5 µM ODS 100×4.6 mm column maintained at 37°C. The mobile phase was methanol-buffer (40∶60, v/v), the buffer being a 50 mM anhydrous solution of potassium monobasic phosphate at pH 6.8 (adjusted using 5 M potassium hydroxide solution), running isocratically over 3.5 min at a flow rate of 1 ml.min^−1^. Data were collected using a fluorescence detector (RF2000; Dionex) set at 515 nm (excitation) and 553 nm (emission). For calibration a standard curve was prepared using a TEP stock solution (5 µM in 40% ethanol) serially diluted using 40% ethanol. TEP standards were assayed in triplicate and showed high repeatability (r = 0.99, P<0.0001, n = 12). SL did all analyses blindly with respect to treatments.

### Plasma lipid peroxidation

The MDA levels in plasma were assessed following the same procedure as for sperm samples (see above) except that samples were directly used and did not require the first sonication step.

### KRL test

We also assessed male resistance to oxidative stress using the KRL test (Brevet Spiral V02023, Couternon, France; http://www.nutriteck.com/sunyatakrl.html) adapted to physiological parameters of birds (osmolarity, temperature) [52], [53]. This assay reflects the current availability of total antioxidant defenses (enzymatic and non-enzymatic) as well as the past oxidative insults experienced by red blood cells [54], [55] and also indicates the rates of lipid peroxidation in the erythrocyte membrane [56]. Briefly, 7 µl of whole blood were immediately diluted in 255.5 µl of KRL buffer (150 mM Na, 120 mM Cl^−^, 6 mM K^+^, 24 mM HCO_3_^−^, 2 mM Ca^2+^, 340 mOsM, pH 7.4) and stored at 4°C before analysis 6.2±3 hours after blood collection. The time elapsed before performing the analyses did not influence the results (F _1, 27_ = 0.86, p = 0.36). We loaded 80 µl of KRL-diluted whole blood into wells of a 96-well microplate. We subsequently added to each well 136 µl of a 150 mM solution of 2,2′-azobis-(amidinopropane) hydrochloride (AAPH; a free radical generator), that is, 646 mg of AAPH diluted in 20 ml of KRL buffer [57]. The microplate was subsequently read with a microplate reader spectrophotometer (PowerWave XS reader, Witec AG, Switzerland) at 40°C. The rate of haemolysis was determined by the change in optical density measured at 540 nm [52], [53], [58]. Because of technical reasons, only a subsample of males could be assessed for this assay.

### Statistical analyses

To account for initial values and because we were interested in within-male variation according to treatments, we conducted a restricted maximum-likelihood generalized linear mixed-effects model (REML-GLMM with normal distribution of the error) for repeated measures to analyze the effect of the treatments on sperm swimming velocity and percentage of motile sperm (mean over the three measurement times: 0, 60 and 120 seconds after ejaculation) between day 7 and day 13 post-hatch, including the male identity as a random factor and the day of capture as a fixed factor (Table 1). We further performed repeated models to analyze the effect of the treatments on 1) the decline in sperm swimming velocity and 2) the decline in percentage of motile sperm at the start of the experiment (7 days) and six days post-manipulation (13 days post-hatch) separately, including the male identity as a random factor and time after ejaculation as a fixed factor (Table 2). We used the values immediately after ejaculation (time = 0) to run repeated models to analyze the effect of treatments on MDA levels in sperm, MDA levels in plasma and KRL, all log-transformed to normalize data, between days 7 and 13 post hatch (Table 3), including the male identity as a random factor and the day of capture as a fixed factor. The explanatory variables used in these models were the immune challenge treatment, the vitamin E supplementation, the breast color (PCA scores), and their two-way interactions. We also conducted separate linear models on days 7 and 13 to test for an influence of the MDA levels in sperm on sperm velocity and motility. In all models brood size at hatching and laying date were entered as covariates. Following recent recommendations [59], we did not conduct stepwise reduction procedures and thus report initial, full models.

**Table 1:** REML-GLMM for repeated measures testing for a within-male effect of the immune challenge and the vitamin E-supplementation on mean sperm swimming velocity and mean percentage of motile sperm (means over the three measurement times) between day 7 and 13 post-hatch.

	Sperm swimming velocity			Percentage of motile sperm		
Effect	Estimate ± s.e	*F*_df_	*P*	Estimate ± s.e	*F*_df_	*P*
(Intercept)	15.83±13.25	-	-	0.26±0.14	-	-
Laying date	−0.05±0.19	0.08_1, 64_	0.78	0.00±0.00	1.27_1, 64_	0.26
**Immune Challenge** a	**7.08±3.38**	**4.38_1, 64_**	**0.04**	0.02±0.03	0.85_1, 64_	0.36
Vitamin E supplementation^b^	2.84±3.35	0.72_1, 64_	0.40	−0.04±0.03	3.00_1, 64_	0.09
**Day** c	**7.46±3.28**	**5.17_1, 49_**	**0.03**	0.00±0.03	0.01_1, 49_	0.94
Initial brood size	0.48±0.67	0.52_1, 64_	0.47	0.00±0.01	0.45_1, 64_	0.51
Breast color	1.85±1.41	1.73_1, 64_	0.19	−0.01±0.01	0.44_1, 64_	0.51
Vitamin E supplementation^b^×Day^c^	−4.32±3.62	1.42_1, 49_	0.24	0.00±0.03	0.00_1, 49_	0.96
Immune Challenge^a^×Vitamin E supplementation^b^	−4.92±3.76	1.71_1, 64_	0.20	−0.01±0.03	0.06_1, 64_	0.81
Immune Challenge^a^×Day^c^	−6.36±3.63	3.06_1, 49_	0.09	0.02±0.01	2.30_1, 49_	0.13
Immune Challenge^a^×Breast color	−1.64±1.24	1.77_1, 64_	0.19	0.00±0.01	0.05_1, 64_	0.83
Vitamin E supplementation^b^×Breast color	−2.21±1.19	3.46_1, 64_	0.07	0.00±0.01	0.17_1, 64_	0.68
**Breast color×Day** c	**2.5±1.11**	**5.03_1, 49_**	**0.03**	0.00±0.00	1.27_1, 49_	0.26

Significant terms in the model are highlighted in bold.

a Relative to the PBS-injected group.

b Relative to the placebo group.

c Relative to first capture on day 7.

**Table 2:** REML-GLMM for repeated measures testing the effect of the immune challenge and the vitamin E-supplementation on dynamics of sperm velocity (measured 0, 60 and 120 seconds after ejaculation) on day 13 post-hatch.

	Sperm swimming velocity			Percentage of motile sperm		
Effect	Estimate ± s.e.	*F*_df_	*P*	Estimate ± s.e	*F*_df_	*P*
(Intercept)	9.27±17.80	-	-	0.25±0.25	-	-
Laying date	0.18±0.26	0.47_1, 56_	0.5	0.003±0.004	0.96_1, 56_	0.33
Immune Challenge^a^	−0.41±4.10	0.01_1, 56_	0.92	−0.004±0.05	0.00_1, 56_	0.94
Vitamin E supplementation^b^	1.18±3.94	0.09_1, 56_	0.77	−0.06±0.06	1.3_1, 56_	0.26
**Time**	**-**	**11.85_2, 111_**	**<0.01**	**-**	**53.12_2, 111_**	**<0.01**
Initial brood size	1.11±0.91	1.49_1, 56_	0.23	0.01±0.01	1.3_1, 56_	0.26
**Breast color**	**2.65±1.00**	7.07_1, 56_	**0.01**	0.01±0.01	1.05_1, 56_	0.31
Immune Challenge^a^×Vitamin E supplementation^b^	−1.54±5.00	0.09_1, 56_	0.76	0.00±0.07	0.00_1, 56_	0.99
Time×Breast color	-	0.3_2, 111_	0.74	-	1.16_2, 111_	0.32
**Time×Immune Challenge** a	-	3.23_2, 111_	**0.043**	-	0.45_2, 111_	0.64
Time×Vitamin E supplementation^b^	-	2.22_2, 111_	0.11	-	0.81_2, 111_	0.45

Significant terms in the model are highlighted in bold.

a Relative to the PBS-injected group.

b Relative to the placebo group.

**Table 3:** REML-GLMM for repeated measures testing for a within-male effect of the immune challenge and the vitamin E-supplementation on MDA levels in semen, MDA levels in plasma and KRL between day 7 and 13 post-hatch.

	MDA levels in sperm			MDA levels in plasma			KRL		
Effect	Estimate ± se	*F*_df_	*P*	Estimate ± s.e	*F*_df_	*P*	Estimate ± s.e	*F*_df_	*P*
(Intercept)	3.53±0.94	-	-	1.34±0.64	-	-	1.96±1.12	-	-
Laying date	−0.01±0.01	0.32_1, 62_	0.58	0±0.01	0.17_1, 64_	0.68	0±0.02	0.04_1, 60_	0.84
Vitamin E supplementation^b^	−0.17±0.22	0.57_1, 62_	0.45	−0.07±0.15	0.22_1, 64_	0.64	−0.38±0.24	2.51_1, 60_	0.12
**Day** c	−0.1±0.22	0.22_1, 40_	0.64	−0.21±0.12	2.85_1, 40_	0.10	**−0.81±0.25**	**10.36_1, 22_**	**<0.01**
Immune Challenge^a^	0.01±0.23	0.00_1, 62_	0.95	0.06±0.15	0.14_1, 64_	0.71	−0.2±0.24	0.65_1, 60_	0.42
**Initial brood size**	0.03±0.05	0.33_1, 62_	0.57	**−0.06±0.03**	**3.89_1, 64_**	**0.05**	0.05±0.05	1.06_1, 60_	0.31
Breast color	0.07±0.06	1.41_1, 62_	0.24	0.02±0.03	0.42_1, 64_	0.52	−0.04±0.06	0.44_1, 60_	0.51
**Day** c **×Vitamin E supp.** b	−0.03±0.25	0.02_1, 40_	0.89	0.02±0.13	0.03_1, 40_	0.87	**0.7±0.28**	**6.39_1, 22_**	**0.019**
Day^c^×Immune Challenge^a^	0.01±0.25	0.00_1, 40_	0.97	0.00±0.13	0_1, 40_	0.98	0.04±0.29	0.02_1, 22_	0.9
Day^c^×Breast color	−0.15±0.08	3.53_1, 40_	0.07	−0.07±0.04	2.64_1, 40_	0.11	0.02±0.09	0.04_1, 22_	0.85
Immune Ch.^a^×Vit. E supp.^b^	−0.27±0.25	1.19_1, 62_	0.28	0.1±0.18	0.3_1, 64_	0.59	0.41±0.28	2.16_1, 60_	0.15

Significant terms in the models are highlighted in bold.

a Relative to the PBS-injected group.

b Relative to the placebo group.

c Relative to first capture on day 7.

All analyses were performed with R 2.11.1 and the “nlme” package [60]. Tests are two-tailed with a significance level set to α = 0.05. Sample sizes vary from one analysis to another because not all individuals could be measured for all traits.

## Results

**Figure 1:**
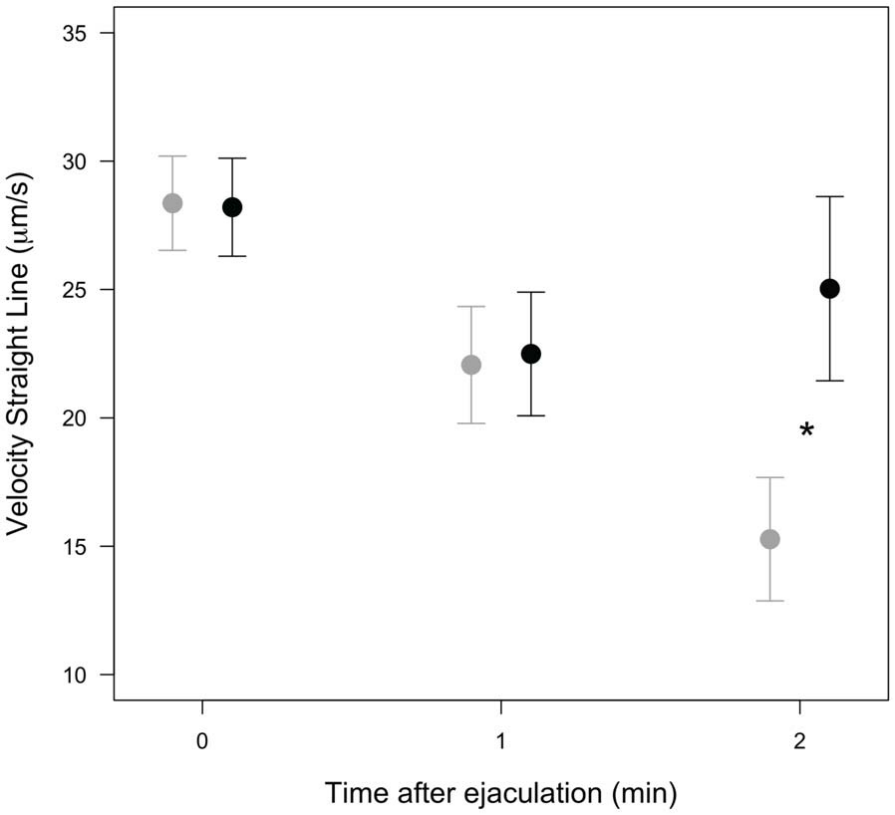
Sperm swimming velocity (mean ± se) in relation to the time after ejaculation and the immune challenge. LPS-injected males: grey dots; PBS-injected males: black dots. Immune-challenged males showed significantly reduced sperm velocity two minutes after ejaculation (Tukey-adjusted post-hoc test: z = 2.74, p = 0.036).

**Figure 2:**
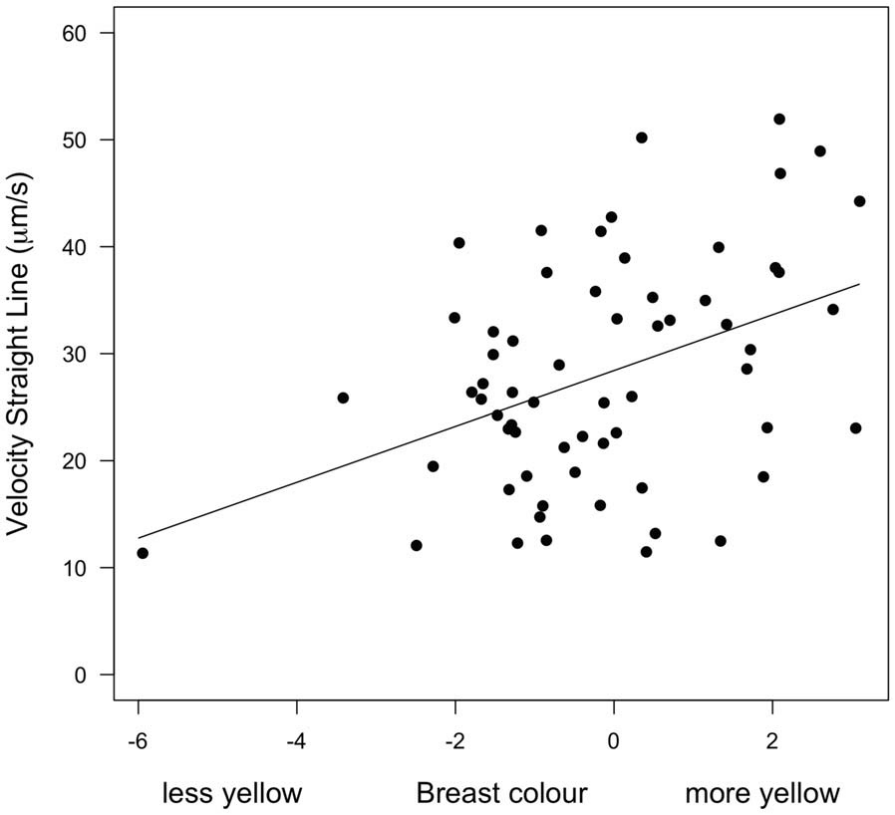
Sperm swimming velocity 13 days post-hatch in relation to plumage breast coloration (PCA scores). The line is the linear regression line. Excluding the outlier from the analysis did not qualitatively change the results.

The mean sperm swimming velocity was significantly influenced by an interaction between day of capture and breast color (Table 1) and also marginally non-significantly influenced by the interaction between immune challenge and day of capture (Table 1). Six days after LPS or PBS injection (13 days post-hatch), mean sperm swimming velocity tended to be lower for immune-challenged males. We then analyzed sperm dynamics over time separately for day 7 and day 13. Initial values of sperm velocity before experimental manipulation (7 days post-hatch) did not significantly differ between experimental groups and showed no correlation with any of the covariates (all F_1, 118_<1.28, p>0.26). Only time after ejaculation significantly influenced sperm swimming velocity (F_2, 118_ = 14.19, p<0.001), with sperm velocity declining between 0 and 120 seconds after ejaculation. Six days after LPS or PBS injection (13 days post-hatch), sperm velocity declined more steeply over time in immune-challenged males (Table 2 and Figure 1, see legend for post-hoc tests). The analysis also showed significant, although weak, positive correlation between breast color and sperm velocity (Table 2, Figure 2).

The percentage of motile sperm was not significantly affected by the treatments or by any variable and there was no significant effect of vitamin E treatment on any sperm trait (Table 1). Before our experimental manipulation, the temporal dynamics of the percentage of motile sperm were similar in both groups, and only laying date and time after ejaculation showed significant effects (F_1, 56_ = 5.50, p = 0.02 and F_2, 114_ = 44.83, p<0.001, respectively). Six days post-injection, the temporal dynamics in the percentage of motile sperm did not differ between LPS- and PBS-injected males (Table 2).

**Figure 3:**
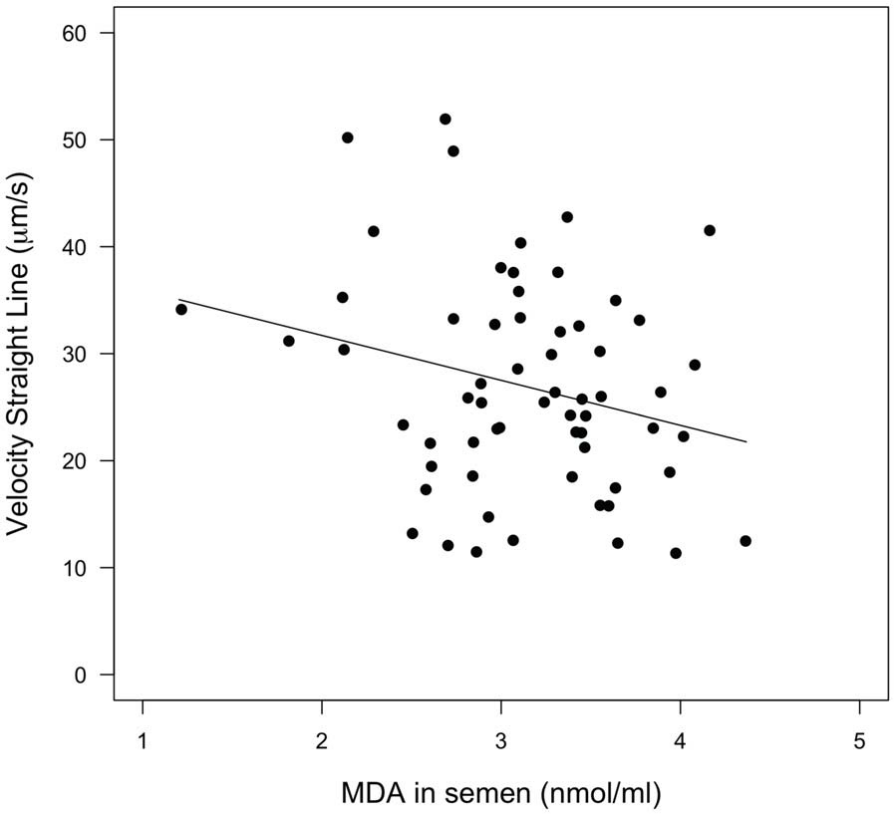
Sperm swimming velocity on day 13 post-hatch in relation to levels of malondialdehyde (end-product of lipid peroxidation) in sperm. The line is the linear regression line.

Sperm swimming velocity was negatively, although weakly, correlated with the MDA levels in sperm six days post-injection (13 days post-hatch) (F_1, 60_ = 4.11, p = 0.047, Figure 3) but not at the start of the experiment (7 days post-hatch) (F_1, 55_ = 0.16, p = 0.69), and no pattern was found for the percentage of motile sperm on either of these days (F_1, 55_ = 0.35, p = 0.56; F_1, 60_ = 0.11, p = 0.74). Finally, MDA levels in sperm and in plasma remained unaffected by our treatments but whole blood resistance to oxidative stress (KRL) was significantly positively influenced by the vitamin E supplementation (Table 3). Vitamin E supplemented males showed a higher whole blood resistance to oxidative stress at the end of the experiment (day 13) (half-life in minutes: 11.48±0.99 and 6.46±0.48, respectively, F_1, 35_ = 9.04, p = 0.005) while males of the two groups did not differ initially (day 7) (13.91±1.08, and 13.83±0.83, respectively, F_1, 48_ = 1.20, p = 0.28).

## Discussion

To our knowledge, this is the first study in a wild, free-ranging bird species showing a cost of immune activation on sperm swimming velocity, a trait likely determining male fertilizing efficiency and sperm competitive ability [19]. However, our results do not support the hypothesis that immune activation generates systemic oxidative stress leading to oxidative damage to sperm. Increasing vitamin E availability to males did also not affect sperm quality and did not help males reduce oxidative damages to sperm. Lastly, we found evidence that male carotenoid-based coloration may advertise sperm swimming velocity, confirming the result of a previous independent study [17].

Sperm swimming velocity, which is positively associated with sperm metabolic performance, predicts the fertilizing efficiency of an ejaculate in both competitive and non-competitive contexts [21], [61], [62]. In birds, sperm swimming velocity progressively decreases after insemination [21] and we found a steeper decline in sperm velocity in males that mounted an immune response. This result has important implications because sperm competition is expected to impose strong selection pressure for producing sperm with high swimming velocity, in particular for species in which females store sperm before fertilization [21]. In birds, spermatozoa are stored in female sperm storage tubules (SSTs) from where they are further continuously lost and moved to the infundibulum, where fertilization occurs [63]. Sperm swimming velocity can be a strong determinant of male fertilizing efficiency because high velocity may predict the number of sperm that are initially stored in the SSTs [64], [65], and high-velocity sperm may remain longer in the storage organ. Thus, as time goes, high-velocity sperm would gain advantage by the number as suggested by recent models and experiments [19], [66], [67]. Our results thus suggest that mounting an immune response can lower male reproductive success, through an increased risk of unfertilized eggs and reduced sperm competitive ability and paternity.

Similarly to previous studies [17], [68], we found levels of oxidative damage in sperm (sperm MDA) to affect sperm quality (sperm swimming velocity). However, this relationship was absent at the start of the experiment. This suggests that lipid peroxidation in sperm membrane may only weakly affect sperm swimming velocity. Oxidative stress has been shown to disrupt proper mitochondria functioning [69]. Thus, measures of *in situ* production of ROS or of the redox balance inside the mitochondria may better predict sperm swimming velocity. Other types of oxidative damage in sperm that we did not measure, such as damages to nuclear DNA, which encodes for most of the proteins involved in mitochondrial functions, may also affect sperm swimming velocity [70]. Finally, although oxidative damage to sperm membrane may only weakly affect sperm velocity, lipid peroxidation in sperm membrane may still have dramatic consequences for male fertility because membrane integrity is crucial for successful sperm-egg interactions and successful fertilization [15], [16].

LPS injection is known to trigger an acute phase response (acute inflammation), which lasts 24 to 72 hours and can result in systemic oxidative stress [14], further leading to an oxidative imbalance in the testes either due to an increase of pro-oxidant compounds or a reallocation of antioxidant defenses to somatic protection and maintenance [15], [71]. Under such conditions, future spermatozoa may be expected to suffer greater oxidative insults all along the spermatogenesis cycle. Information on spermatogenesis in birds comes from a few non-passerine species and show that spermatogenesis lasts between 11 and 14 days [72], [73], [74]. A histological study in the yellow-throated sparrow suggests that spermatogenesis may be shorter in Passerine birds because spermiogenesis comprises fewer steps than in the non-passerine species for which the information is available [75]. We have recaptured males 6 days after we applied our treatments. Therefore, our treatments may be expected to affect future spermatozoa at any stage during the last 6 days of spermatogenesis (i.e. during spermiogenesis which is when spermatozoa acquire motility [75] or spermiation which is when mature spermatozoa are released).

Here, 6 days following the LPS-injection we found no decrease in blood antioxidant resistance, no increase in somatic oxidative damage (plasma levels of MDA) and no increase in sperm oxidative damage (ejaculate levels of MDA). Our results thus provide no direct support for a role of systemic or local oxidative stress in the steeper decline in velocity observed in LPS-treated males. These results contrast with studies showing that even mild inflammation by LPS, an endotoxic component of the gram-negative bacteria cell wall known to activate an acute inflammatory response [76], can disrupt spermatogenesis and affect sperm quality via both up-regulation of inflammatory modulators (e.g. cytokines) and systemic as well as local oxidative stress resulting from increased production of RNOS (reactive nitrogen and oxygen species) by macrophages [15], [77], [78]. However, following LPS-injection, sperm quality has been reported to be maximally altered at the end of the spermatogenesis cycle, suggesting that germ cells at the earliest stage of spermatogenesis (spermatogonia) are more susceptible to immune-induced oxidative stress [77]. Therefore, in the present study, by sampling males 6 days after the acute inflammation, we might have been unable to monitor the bulk of reduction in sperm quality due to immune-induced oxidative stress. Hence, the possibility that germ cells suffered insults by inflammatory RNOS cannot be discarded and deserves to be further investigated by monitoring sperm quality over the entire spermatogenesis cycle.

Nevertheless, because our results cannot be interpreted in light of a direct effect of systemic or local oxidative stress, we should consider alternative hypotheses. We can think of at least two non-mutually exclusive hypotheses to explain the steeper decline in sperm velocity in LPS-injected males. First, inflammation has been shown to impair the proper functioning of Leydig cells leading to disturbed testes functions, particularly steroidogenesis and thereby spermatogenesis, resulting in higher levels of necrotic and apoptotic spermatozoa in the semen of rats and rabbits [77], [79], [80]. Second, since mounting an immune response is energetically costly [2], [8], the steeper decline in sperm velocity observed in LPS-injected males may be due to fewer resources being available for spermatogenesis and sperm quality control following reallocation of energy towards immunity. Whatever the underlying mechanisms, our result nevertheless suggests that sperm quality, measured here as sperm swimming velocity, is a condition-dependent trait, a prerequisite to the phenotype-linked fertility [81] and the good-sperm [82] hypotheses.

Surprisingly, although vitamin E is known to improve sperm quality through a reduction of oxidative stress [25], [83], [84], [85], [86], our supplementation did not help males reduce sperm oxidative damage. Considering the oxidative cost of reproduction [52], [87], [88], internal reserves of dietary antioxidants may be greatly reduced during the nestling rearing period. Thus, one explanation for the absence of an effect of vitamin E on sperm oxidation and quality is that supplementary vitamin E has been devoted to the antioxidant protection of somatic functions related to reproductive effort, as suggested by the contribution of vitamin E to whole-blood resistance to oxidative stress, rather than to antioxidant defense in semen.

In this study, more colorful males produced sperm with greater swimming velocity on day 13 post-hatch. This result confirms previous studies, which demonstrated that male colorful ornaments signal sperm quality [17], [89], [90], [91]. Interestingly, this relationship between carotenoid-based color and sperm quality was revealed at the second capture (13 days post-hatch) while initially absent (7 days post-hatch). Similarly, previous work found a significant relationship between breast color and resistance to oxidative stress towards the end of the rearing period (15 days post-hatch only) while no such relationship was detected before (8 days post-hatch [92]). The second part of the rearing period, i.e. 11 days post-hatch onwards, is thought to be a greatly demanding period in terms of nestling energy requirements and feeding rates [93]. Thus, the fact that the degree of ornamentation correlates with sperm quality when measured during this particularly stressful period suggests that carotenoid-based colors reliably signal a male's ability to protect his sperm when facing an acute stress. The evolutionary relevance of this signal should however reach a maximum during the pre-laying period, when females seek males with high fertilizing potential. The laying period during which females are fertile, lasts for about two weeks and potentially covers a full spermatogenesis cycle [72], [73], [74]. This period may be long enough for males to experience various stresses including oxidative stress. Hence, if females were using male plumage coloration to choose their social or extra-pair partners, they would potentially gain direct (increased fertility) and/or indirect (by producing sons with higher ability to protect their sperm) benefits, because brighter males appear to advertise their ability to produce faster sperm under stressful circumstances. Such a relationship between ornaments and sperm quality is a premise to the phenotype-linked fertility hypothesis [81] and its extensions [94], [95].

To conclude, our results provide evidence that the activation of an immune response incurs costs to males in terms of sperm quality, and further suggest that temporal dynamics in sperm velocity is a condition-dependent trait. We also found that carotenoid-based coloration reflects sperm quality under stressful circumstances. These results have important implications for the evolution of mate choice and multiple mating in females because females may accrue both direct and indirect benefits by mating with males having better quality sperm.
